# Use and Effect of Web-Based Embodied Conversational Agents for Improving Eating Behavior and Decreasing Loneliness Among Community-Dwelling Older Adults: Protocol for a Randomized Controlled Trial

**DOI:** 10.2196/22186

**Published:** 2021-01-06

**Authors:** Lean L Kramer, Bob C Mulder, Lex van Velsen, Emely de Vet

**Affiliations:** 1 Consumption and Healthy Lifestyles Wageningen University & Research Wageningen Netherlands; 2 Strategic Communication Wageningen University & Research Wageningen Netherlands; 3 eHealth Cluster Roessingh Research and Development Enschede Netherlands; 4 Biomedical Signals and Systems Group University of Twente Enschede Netherlands

**Keywords:** embodied conversational agent, health behavior change, loneliness, eating behavior, older adults

## Abstract

**Background:**

An unhealthy eating pattern and loneliness negatively influence quality of life in older age. Embodied conversational agents (ECAs) are a promising way to address these health behaviors in an engaging manner.

**Objective:**

We aim to (1) identify whether ECAs can persuade community-dwelling older adults to change their dietary behavior and whether ECA use can decrease loneliness, (2) test these pathways to effects, and (3) understand the use of an ECA.

**Methods:**

The web-based eHealth app PACO is a fully automated 8-week intervention in which 2 ECAs engage older adults in dialogue to motivate them to change their dietary behavior and decrease their loneliness. PACO was developed via a human-centered and stakeholder-inclusive design approach and incorporates Self-determination Theory and various behavior change techniques. For this study, an unblinded randomized controlled trial will be performed. There will be 2 cohorts, with 30 participants per cohort. Participants in the first cohort will immediately receive the PACO app for 8 weeks, while participants in the second cohort receive the PACO app after a waiting-list condition of 4 weeks. Participants will be recruited via social media, an online panel, flyers, and advertorials. To be eligible, participants must be at least 65 years of age, must not be in paid employment, and must live alone independently at home. Primary outcomes will be self-assessed via online questionnaires at intake, control, after 4 weeks, and after 8 weeks, and will include eating behavior and loneliness. In addition, the primary outcome—use—will be measured via data logs. Secondary outcomes will be measured at the same junctures, via either validated, self-assessed, online questionnaires or an optional interview.

**Results:**

As of July 2020, we have begun recruiting participants.

**Conclusions:**

By unraveling the mechanisms behind the use of a web-based intervention with ECAs, we hope to gain a fine-grained understanding of both the effectiveness and the use of ECAs in the health context.

**Trial Registration:**

ClinicalTrials.gov NCT04510883; https://clinicaltrials.gov/ct2/show/NCT04510883

**International Registered Report Identifier (IRRID):**

PRR1-10.2196/22186

## Introduction

### Background

Unhealthy eating and loneliness negatively influence quality of life (QoL) in older age [1,2]. Statistics show that in the Netherlands, almost 60% of people over 65 are obese [3], and 57% of community-dwelling older adults are at risk for undernutrition [4]. Both are important risk factors for chronic diseases and are clearly associated with unhealthy eating behaviors. As eating is regarded as a social activity, loneliness is associated with a loss of pleasure in eating and cooking [5] and is a significant predictor of malnutrition [6]. Loneliness can be defined as the discrepancy between a person’s desired and achieved levels of social relationships [7]. In the Netherlands, over 50% of older adults indicate that they experience loneliness, a percentage that is even higher among people without a partner [8]. The expected increase in the coming years [9] in this group of single, community-dwelling older adults will exacerbate this problem. However, it is challenging to realize an actual change in eating behavior and deal with loneliness.

Embodied conversational agents (ECAs) have been proposed as a promising technological tool to persuasively address these health behaviors, with the aim of changing users’ attitudes or behaviors through persuasion and social influence rather than through coercion [10]. ECAs can be defined as “more or less autonomous and intelligent software entities with an embodiment used to communicate with the user” [11]. A typical user interface consists of a human-like ECA with prewritten dialogues, including multiple choice answer options [12]. ECAs can make an intervention for coaching people in a healthy lifestyle more engaging than traditional electronic health interventions [12]. This ability is often ascribed to ECAs’ capacity to establish and maintain an emphatic relationship [12,13]. Early studies show that older adults who interact with an ECA form a relationship with the ECA and consider it a companion [14], including those from populations in which eHealth literacy is generally lower [15]. ECAs are perceived as enjoyable, usable, and acceptable for addressing health behavior change [16-18].

Nonetheless, interventions with an ECA are not immune to declining use over time, meaning that this issue must be addressed in ECA design to prevent limited long-term health effects [12]. Furthermore, and even more importantly, evidence of ECA effectiveness and underlying working mechanisms is scarce and remains inconclusive [12]. This limits the possibility to learn from others’ efforts and prevents knowledge accumulation.

### Objectives

We present the protocol for an 8-week evaluation of the PACO service. Consisting of 2 ECAs, PACO is a web-based app that aims to achieve dietary behavior change and decrease loneliness among single, community-dwelling older adults. The goal of the evaluation is to (1) identify whether ECAs can persuade community-dwelling older adults to change their dietary behavior and decrease their loneliness, (2) assess the pathways to effects, and (3) understand ECA use. The latter 2 goals will allow us to explain the occurrence (or the lack) of an effect from using the intervention and can therefore serve to support the design of future ECAs.

### Conceptual Models

**Figure 1:**
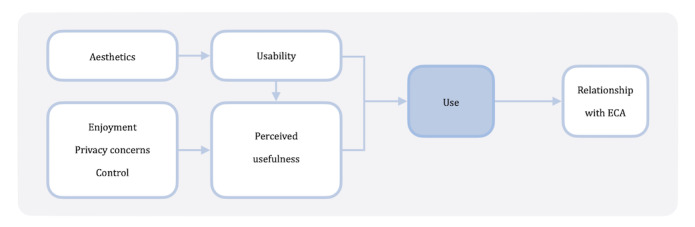
Conceptual model explaining embodied conversational agents (ECA) use, known as CEU.

**Figure 2:**
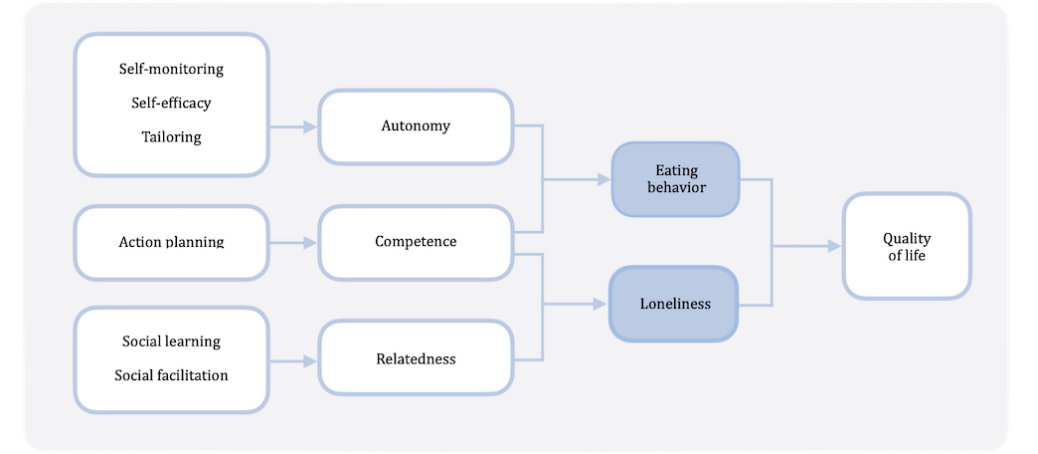
Conceptual model explaining health effects, known as CHE.

In order to conceptualize and measure engagement, Cole-Lewis et al [19] state that it is necessary for users to have appropriate levels of interaction with the technology and that the behavioral change components are relevant. Hence, we present 2 conceptual models. The first is a conceptual model explaining ECA use (CEU; Figure 1). With this model, we aim to explain the factors that determine the use of an ECA intervention in this context. The second is a conceptual model explaining health effects (CHE; Figure 2). With this model, we aim to explain the mechanisms behind the observed change in eating behavior and loneliness.

Use is at the center of the conceptual model explaining ECA use. It is assumed that an eHealth intervention will not be used if it does not create any benefit (perceived usefulness) or if it has a substantial number of usability problems [20]. Visual aesthetics, defined as an orderly and clear design, are closely related to many of the design rules advocated by usability experts [21]. In the case of patient portals, aesthetics have thus been found to influence usability in the context of explaining technology acceptance [22]. We expect perceived usefulness to be influenced by 3 user experience factors. Perceiving something as enjoyable is linked to a positive effect on use when the system is perceived to be useful [23]. Willingness to share personal information and preferences (ie, the absence of privacy concerns) is argued to be a prerequisite for convenience and a useful system [24]. The last factor is control, which refers to “the extent to which the user can bring about or prevent particular actions or states of the system if she has the goal of doing so” [25]. Especially in human-computer interaction literature, control has been identified as a crucial factor in the occurrence of perceived usefulness and use [26,27]. Furthermore, there is robust evidence that usability has a direct effect on perceived usefulness [28]. In turn, use is hypothesized to act as an antecedent of the intensity of an end user’s relationship with an ECA [14].

Self-determination Theory (SDT) comprises the basis of the conceptual model explaining health effects [29]. Briefly, SDT postulates that human beings have 3 essential psychological needs: autonomy (the feeling of being the origin of one’s own behaviors), competence (feeling effective), and relatedness (the need to feel belongingness and connectedness with others). Self-monitoring and self-efficacy are associated with increased autonomy [30]. Tailoring is a more generic behavior change technique (BCT), which, in our case, refers to a tailored recipe book. We hypothesize that the possibility of generating a tailored recipe book leads to an increased feeling of being in control. Action planning is found to be supportive of increasing competence [31]. Both social learning and social facilitation are expected to lead to an increase in relatedness, as they both connect people. In turn, a decrease in loneliness and an improvement in eating behavior is expected to lead to more positive health-related QoL outcomes [1,2].

### Research Questions

Our research questions focus on both use and health outcomes. The research questions related to use are as follows: (1) What factors affect the use of the ECA? (2) Does use affect the users’ relationship with the ECA? (3) What is the use of PACO over time? (4) How do users experience PACO use? We will test the CEU to answer research question (RQ) 1 and 2. Via data log analyses (RQ3) and interviews (RQ4) we aim to explain the findings related to the CEU. This way, we will triangulate results.

The research questions related to health effects are as follows: (1) To what extent does PACO reduce loneliness and improves eating behavior and, ultimately, QoL? (2) To what extent does PACO increase autonomy, competence, and relatedness? (3) How does PACO use affect the loneliness and eating behavior of older adults? We will test the CHE to answer RQ1. For RQ2 and RQ3, we will compare the effect of using PACO at different time points, including control.

## Methods

### Study Design

**Figure 3:**
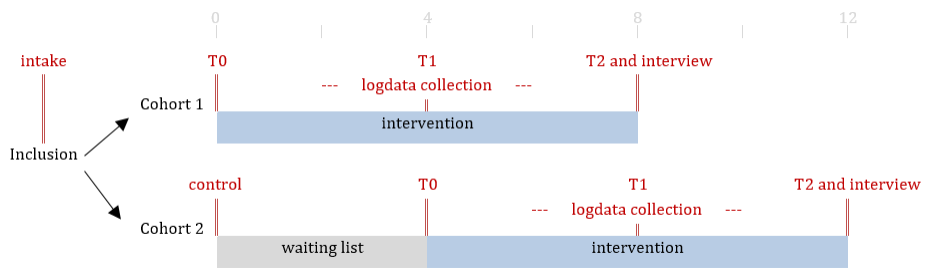
Study design.

An unblinded randomized controlled trial will be carried out. At the time of study protocol submission, all preparations have been made to start recruitment. There will be 2 cohorts (Figure 3). Participants in the first cohort will receive the 8-week PACO app immediately. Participants in the second cohort will receive the PACO app after a 4-week waiting-list condition and serve as a control group. A combination of various data collection methods will be used for this study, including questionnaires (control, at intake, T0, T1, and T2), data log collection during the intervention period, and an optional interview afterward. The T0 questionnaire will mark the start of the intervention, T1 will be completed after 4 weeks of use, and T2 after 8 weeks of use (Figure 3).

### Participants

We aim to include a total of 60 participants: 30 in cohort 1 and 30 in cohort 2. The number of participants is based on the 10-times rule, a widely used minimum-sample-size estimation method for partial least squares structural equation modeling (PLS-SEM) [32]. This method was discussed with a statistician. In addition, we considered the current practices in the field [12], the explorative nature of this study, and the staff available to provide support.

Participants will be considered eligible if they are aged 65 years or older, are not in paid employment, and live alone independently at home. These inclusion criteria fit a potential target audience of almost 1 million people [33]. In addition to these criteria, participants need to speak Dutch to use the app, should be able to use a tablet or computer by themselves, and should have a wireless internet connection at home, which is required for the app. The latter 2 criteria seem feasible, as 94.5% of all older Dutch adults aged 65-75 years have internet access at home, with 77% using the internet daily [34]. Apart from willingness to provide informed consent, there are no exclusion criteria.

### Recruitment

The project members will recruit participants via different routes. An email will be sent out to an existing online panel of older adults (*Ouderenpanel*). Flyers will be distributed in neighborhoods, community centers, sports canteens, and other settings frequently attended by older adults. Advertorials will be placed in local and community newspapers and on social media. Both the flyers and the advertorials will contain a short link to the PACO website with more information and the form to sign up. Lastly, participants will be encouraged to invite relatives.

### Procedure

Interested people can visit a website to view more information, or they can contact the researchers. People can choose to receive the information letter and consent form by post (Multimedia Appendix 1) or view and complete the form online. After providing informed consent, participants will be invited to complete the intake questionnaire. In this questionnaire, they will be asked to report their demographics (gender, age, educational level, health conditions, risk of malnutrition [35], and eHealth literacy [36]), whether they own a device to use for the study, and their motivation to participate. A copy of the signed informed consent will be sent to all participants by mail. The researchers will mail people who do not return the informed consent by post to check whether something has gone wrong or they do not wish to participate (no explanation will be required).

After completing both the informed consent and the intake questionnaire, participants will be assigned a random 4-digit research number. To allocate participants to a cohort, they will be randomly assigned a digit, either 1 or 2, in a list generated by Excel (version 16.0.13426.20274; Microsoft). Participants will receive an email from author LK containing their research number, information on their allocated cohort, and a copy of the information letter and the signed informed consent.

Participants in cohort 2 will first be asked to complete the additional control questionnaire (Table 1) and will start the intervention period after 4 weeks. At the start of the intervention period, participants will receive an email with instructions for the onboarding process. The email will contain a video message from the researchers introducing themselves and the project, as well as a link to the freely available PACO website. Once participants have created an account, they will be asked to complete the T0 questionnaire, assessing the health parameters and relationship with the ECA. Participants will be phoned within 2 working days and asked whether they have any questions. If a participant does not wish to be called, this can be indicated by email. If a participant needs help, the researchers will offer to visit the participant. A logbook will be kept of all such contacts. If participants do not have a tablet or a computer, they will be given a tablet for the duration of the study.

**Table 1:** Metadata and factors measured via questionnaires, per study phase.

Metadata and factors			Questionnaires						
			Control		T0		T1		T2
**Metadata questionnaire**									
	Total number of questions, n	46		56		65		88	
	Minutes to complete	10 -20		15-20		15-20		25-30	
**Conceptual model explaining health effects**									
	Eating behavior	✓		✓		✓		✓	
	Loneliness	✓		✓		✓		✓	
	Autonomy, competence, and relatedness	✓		✓		✓		✓	
	Quality of life	✓		✓		✓		✓	
**Conceptual model explaining ECA^a^ use**									
	Relationship with ECA^a^			✓		✓		✓	
	Usability							✓	
	Enjoyment							✓	
	Aesthetics							✓	
	Privacy concerns							✓	
	Control							✓	
	Perceived usefulness							✓	
**Other constructs**									
	User experience					✓			
	Willingness to pay							✓	

^a^ECA: embodied conversational agent.

After 4 weeks of use, participants will receive an email with an invitation to complete the online T1 questionnaire, assessing all their health factors, their relationship with the ECA, and their user experience. After 8 weeks of use, participants will receive an email with an invitation to complete the T2 questionnaire, assessing all their health and use factors and willingness to pay. In addition, participants will be asked whether they are open to an interview lasting half an hour, in which the researcher will ask about their user experience.

At all stages of the study, participants will be able to contact the researchers by email or phone for any questions or problems. In the PACO app, there is a contact form. Depending on the participant’s problem and preference, one of the researchers will email, phone, or visit the participant. If a participant has not interacted with PACO for 7 days, the participant will also be contacted and asked whether there are any problems.

### Intervention

#### PACO App

**Figure 4:**
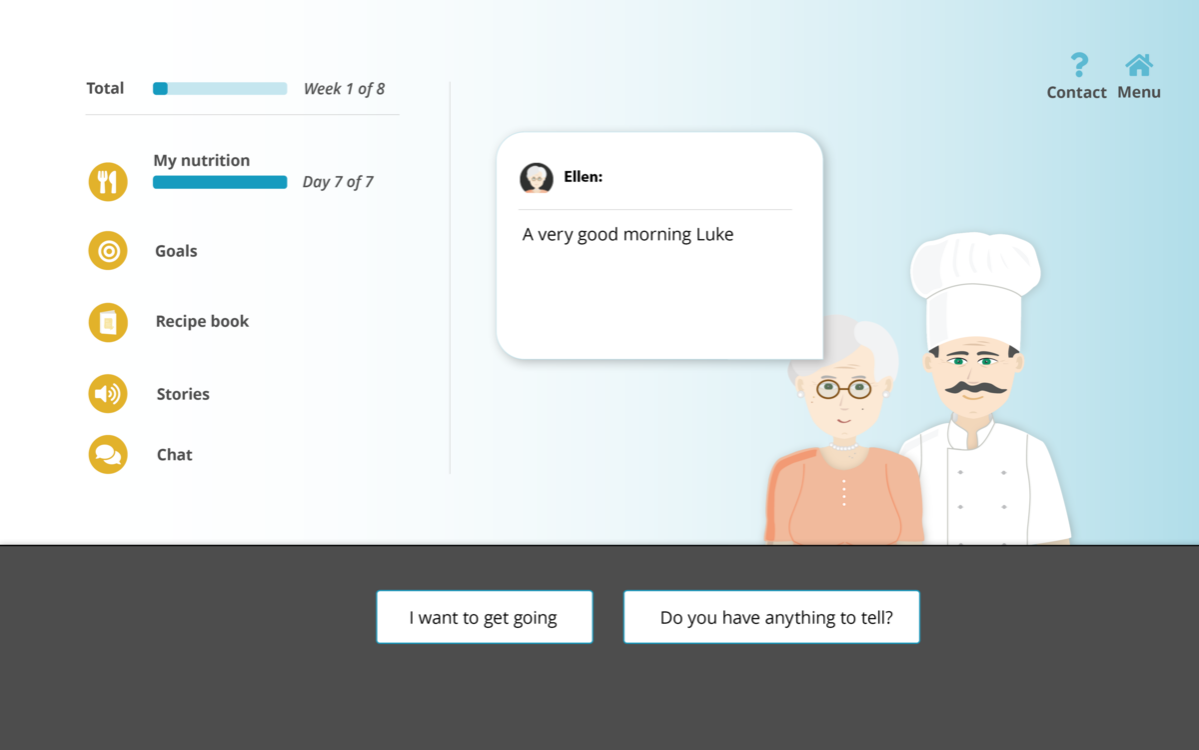
PACO app home screen.

PACO is a fully automated web-based eHealth app in which 2 ECAs engage in dialogue with older adults in order to motivate them to change their dietary behavior and decrease their loneliness (Figure 4 and Multimedia Appendix 2). The app consists of 5 modules, each one applying different BCTs (Table 2). During the onboarding process, the ECAs introduce themselves and explain the PACO program. There is a daily dialogue between either Herman (the cook, who provides nutritional advice) or Ellen (the peer, who provides social advice) and the user. Users are asked to use the food diary module for the first 7 days in order to increase their awareness of their eating behavior. All other modules become available when the food diary has been completed for 7 days or automatically after 14 days. During and after the 8-week program, the ECAs encourage users to continue the health behavior changes that they have implemented during the intervention in their daily lives.

**Table 2:** The modules of the PACO app.

Week	Module	Behavior change technique	SDT^a^ component, target behavior	Rationale
1	Food diary	Self-monitoring	Autonomy, eating behavior	Users record what they have eaten, with whom, and how they appreciated the meal. There is an option to set reminders. When users know what they eat and drink, we aim to give them the feeling that they are able to change their behavior [37], leading to an actual change in eating behavior.
2-8	Goals	Action planning	Competence, eating behavior and loneliness	Users can choose from a list of social and eating goals. Via dialogue, Ellen explains the goal and provides tips. Users create a personal action plan and track their progress, with the option to set reminders. When users carry out their plans, we aim to improve their feelings of competence [31], leading to changes in eating behavior and feelings of loneliness.
1-8	Recipes	Tailoring and self-efficacy	Autonomy, eating behavior	Via dialogue, Herman helps users select a healthy and easy-to-prepare recipe (>280), based on users’ dietary wishes and preferences. By assisting users in cooking their own meals in line with their preferences, we hope to increase feelings of autonomy via self-efficacy [38], leading to a change in eating behavior.
1-8	Stories	Social learning	Relatedness, loneliness	Users can listen to stories from other older adults about physical or virtual social activities they perform. Ellen can provide more information on the activity. When users learn from each other, we hope that they will feel more related to others and have fewer feelings of loneliness [39,40].
1-8	Chat	Social facilitation (peer support)	Relatedness, loneliness	Via WhatsApp groups, users can interact with one another. Ellen is also included and asks questions. When users interact with one another, we hope that they will experience increased feelings of relatedness and decreased feelings of loneliness [41,42].

^a^SDT: Self-determination Theory.

#### Development of the Intervention

The PACO development process was based on the first 3 steps of the Center for eHealth Research and Disease Management (CeHRes) Roadmap [43]: the contextual inquiry, the value specification, and the design phase. The contextual inquiry phase consisted of 3 parts. First, the current practices in designing and evaluating ECAs for coaching people in the health context were identified via a scoping review [12]. Second, factors contributing to healthy living and healthy and unhealthy eating among Dutch community-dwelling older adults were identified via a 7-day diary and via multiple focus groups [44]. Third, an initial stakeholder analysis was carried out, and key stakeholders were identified [45].

During the value specification phase, healthy eating tips were explored via 2 additional focus group sessions [44]. The preferred approach, source, and tone of voice for healthy eating tips were discussed. In addition to the focus group, interviews were held with key stakeholders in order to identify their requirements [45].

The design phase of the PACO app was based on the previous 2 phases. In addition, the SDT [29] was used as a foundation. Self-monitoring, action planning, tailoring, self-efficacy, social learning, and social facilitation were selected as BCTs. In the design phase, first, 3 ECAs were created, each with a different role. In order to ascertain their persuasiveness, an online experiment using various mockups was carried out. Via a focus group, the findings were discussed, and the layout of the 2 preferred ECAs was improved. All input was used to create a first version of the app, which was tested via a usability study [46]. All usability issues were resolved, leading to the final app.

### Data Collection

The main study parameters include use, eating behavior, and loneliness. Use will be assessed via data logs, which will be collected by the PACO app. More specifically, data logs contain the user ID, timestamp, dialogue, and ECA (either Ellen or Herman). In addition, data logs contain the number of goals achieved, the diary input, and the chat history. Eating behavior will be self-assessed by 3 open questions, based on a 24-hour recall format. The questions include the following: (1) Did you eat fruit yesterday? If so, what kinds of fruit, what time, and how many grams per piece? (2) Did you eat vegetables yesterday? If so, what kinds of vegetables, what time, and how many grams per piece? (3) Did you drink yesterday? If so, what kinds of drink, what time, and how many glasses, cups, or milliliters? Loneliness will be self-assessed via a questionnaire (Table 3).

**Table 3:** Details of the questionnaires.

Factor	Questionnaire	Items, n	Scale	Modifications
Loneliness	De Jong Gierveld Loneliness Scale [47]	6	1-5	None
Autonomy, competence, and relatedness	Basic Psychological Need Satisfaction and Frustration Scales [48-50]	24	1-5	None
QoL^a^	Brief Older People’s Quality of Life [51]	13	1-5	Translated to Dutch
Relationship with ECA^b^	Rapport Scale [52-54]	10	1-5	Translated to Dutch; ‘virtual coach’ instead of ‘coordinator’
Usability	System Usability Scale [55]	10	1-5	Translated to Dutch
Enjoyment	Affect Scale [56]	4	1-5	Translated to Dutch
Aesthetics	Classic Aesthetics [21]	5	1-7	Translated to Dutch
Privacy concerns	Concern for Privacy Scale [24]	4	1-7	Translated to Dutch
Control	Active Control [57]	4	1-7	Translated to Dutch; ‘PACO’ instead of ‘website’
Perceived usefulness	Perceived Usefulness Scale [28,58]	3	1-5	Translated to Dutch; ‘PACO’ instead of ‘the robot’

^a^QoL: quality of life.

^b^ECA: embodied conversational agent.

The secondary study parameters include self-determination (autonomy, competence, relatedness), QoL, relationship with ECA, usability, enjoyment, aesthetics, privacy concerns, control, and perceived usefulness. All these parameters will be measured via validated, self-assessed, online questionnaires.

In addition, 2 other parameters include willingness to pay and user experience. Both parameters will be assessed via self-compiled questionnaires. Participants will be asked whether they are willing to pay for PACO (*yes*/*no*) and the amount they are willing to pay for PACO for 3 months [€0, €5 (USD $6.09), €10 (USD $12.18), or €20 (USD $24.35) per month]. Via a questionnaire, participants will be asked 9 open-ended questions about their user experience in general (eg, How did you experience using PACO the last 4 weeks?) and per module (eg, Which modules did you perceive as useful, and why?). In addition, participants will be asked why they kept using PACO, why they stopped using PACO, and whether they wish to share something else. Via an interview of approximately 30 minutes, participants will be asked about their general experience, the modules, how and where they used PACO, experienced behavior change, and the two coaches. Via these questions, we aim to gain a more fine-grained understanding of users’ experiences and triangulate our quantitative results.

### Data Analysis

Descriptive statistics will be used for participant demographics, data logs, and willingness-to-pay data. Data logs will be used to determine the frequency of login, time spent on each module, time spent in total, time of use, and time of dropout. If a participant has not interacted with PACO for 14 consecutive days, they will be treated as a dropout and omitted from further analysis. The within-subject *t* test will be used to compare effects between control, T0, T1, and T2. PLS-SEM will be used in 2 phases per model to test the conceptual models. In phase 1, the measurement model will be validated by testing the constructs separately to determine internal validity [using structural equation modeling (SEM)-oriented criteria and a traditional Cronbach alpha]. In addition, it will be determined whether there is multicollinearity. If there is an acceptable measurement model, phase 2 will be carried out. The causal model will be tested and, if necessary, optimized. A conservative approach will be adopted whereby the theoretical model will be adjusted only if this results in a large improvement in the model. The quality of the causal model will be determined on the basis of PLS-SEM specific goodness-of-fit indices. All analyses will be performed in SPSS (version 24; IBM Corp) and SmartPLS (version 3; SmartPLS GmbH).

Audio recordings of the interviews will be transcribed until data saturation is reached. The transcripts of, and answers to, the open user-experience questions will be uploaded in ATLAS.ti qualitative data analysis software (version 8.4; ATLAS.ti Scientific Software Development GmbH). Analysis will be guided by a thematic analysis approach [59], combining a deductive and an inductive approach. The protocol for the focus group will be used to generate deductive codes. An initial list of inductive codes will be generated by LK and supplemented independently by another researcher. Differences will be discussed, leading to a final and agreed upon codebook. Each transcript will be coded by LK and another project member. Differences will be discussed again, leading to a final coded transcript.

### Ethical Considerations

The study has been approved by the medical ethical committee of Wageningen University (number NL73121.081.20) and has been registered at ClinicalTrials.gov before the enrolment of participants (identifier NCT04510883). As participants are not exposed to any risks, a data safety monitoring board will not be used during the study. Participants will invest time in this study; they have to complete multiple surveys and use the PACO app for 8 weeks. We believe that this duration and data collection are needed to gain a fine-grained understanding of the app’s use, relationship development, and the process of health behavior change. The main benefit to participants is that they gain insight into their health behavior via the PACO app. In addition, in prior studies, we found that participants highly appreciate the attention given to them via such studies.

Prior to the study, people will receive an information letter. They will have 2 weeks of consideration time and can contact the researcher (LK) or an independent expert with any questions. Participants can leave the study at any time for any reason if they wish to, without any consequences; participants will not be replaced.

All collected data will be kept in secure online databases that are password-protected, with access limited to the study team.

## Results

As of July 2020, we have begun recruiting participants.

## Discussion

### Overview

ECAs seem to be a promising means of addressing health behavior change in general, including among community-dwelling older adults. In this paper, we have described the protocol for an unblinded randomized controlled trial among older adults, with the goal of understanding the use and health effects of ECAs that provide nutritional advice. The intervention at the center of this evaluation is called PACO and provides 2 ECAs, one that offers nutritional advice (a cook) and one that offers social advice (a peer). The intervention was developed via a human-centered and stakeholder-inclusive design approach, incorporating theory and various BCTs. At the time of writing, evidence on the effectiveness and underlying working mechanisms of ECA use for health purposes remains inconclusive [12]. In order to explain the effects (or the absence thereof), we developed 2 conceptual models. The first model explains the use of an ECA intervention, and the other explains the mechanisms behind the observed change in eating behavior and loneliness after using an ECA intervention. Via the randomized controlled trial, both models will be tested, and use, user experience, and potential health effects will be assessed. In this way, we aim to generate insight into the effect and design of ECAs that go beyond the PACO service and that serve the eHealth community in general [60].

### Limitations

Like any evaluation plan, ours has some limitations. The first limitation relates to our recruitment strategy. We expect to include more women than men. In previous studies [44], we found women to be more interested in lifestyle-related studies, resulting in focus group sessions with an overrepresentation of women. However, given that there are more single women than men in older age groups in the Netherlands [61], a majority of women is a realistic reflection of society. Another aspect of our recruitment strategy is that participants are encouraged to invite relatives, and this might induce a risk of contamination. If a closely related person joins the study, we will monitor this meticulously to see whether contamination takes place, and whether and how it influences our results.

Second, use of the ECA intervention might be influenced by the study itself. Participants know that they are expected to complete multiple questionnaires and will be called by phone if they do not use the app for 7 days. Thus, participants might be inclined to use the app more often than if the intervention were applied outside a randomized controlled trial setting. However, in order to understand the determinants affecting use and health effects, the questionnaires are essential, and a phone call is needed to ensure that there are no technical issues influencing use.

### Conclusion

By unraveling the mechanisms behind the use of a web-based service that offers 2 ECAs, we hope to gain a fine-grained understanding of both the effectiveness and the use of ECAs. These insights will boost the design, the use, and the usefulness of ECAs in health behavior change.

## Appendix

Multimedia Appendix 1 Information letter and informed consent.

Multimedia Appendix 2 Peer-review report.

Multimedia Appendix 3 CONSORT-eHEALTH checklist (V1.6.1).

## Abbreviations

BCT: behavior change technique
ECA: embodied conversational agent
CEU: conceptual model explaining ECA use
CHE: conceptual model explaining health effects
PLS-SEM: partial least squares structural equation modeling
QoL: quality of life
RQ: research question
SDT: Self-determination Theory
SEM: structural equation modeling
