# Supplementary material for: Use and Effect of Web-Based Embodied Conversational Agents for Improving Eating Behavior and Decreasing Loneliness Among Community-Dwelling Older Adults: Protocol for a Randomized Controlled Trial
Source: JMIR Res Protoc. 2021 Jan 6;10(1):e22186. doi: 10.2196/22186 (PMC7817356; doi:10.2196/22186)
Supplement: Multimedia Appendix 1 [file resprot_v10i1e22186_app1.pdf]

Proefpersoneninformatie voor deelname aan medisch-wetenschappelijk onderzoek

[PACO evaluatie]

Officiële titel: Gebruik en gezondheidseffecten van Virtuele Coaches onder ouderen.

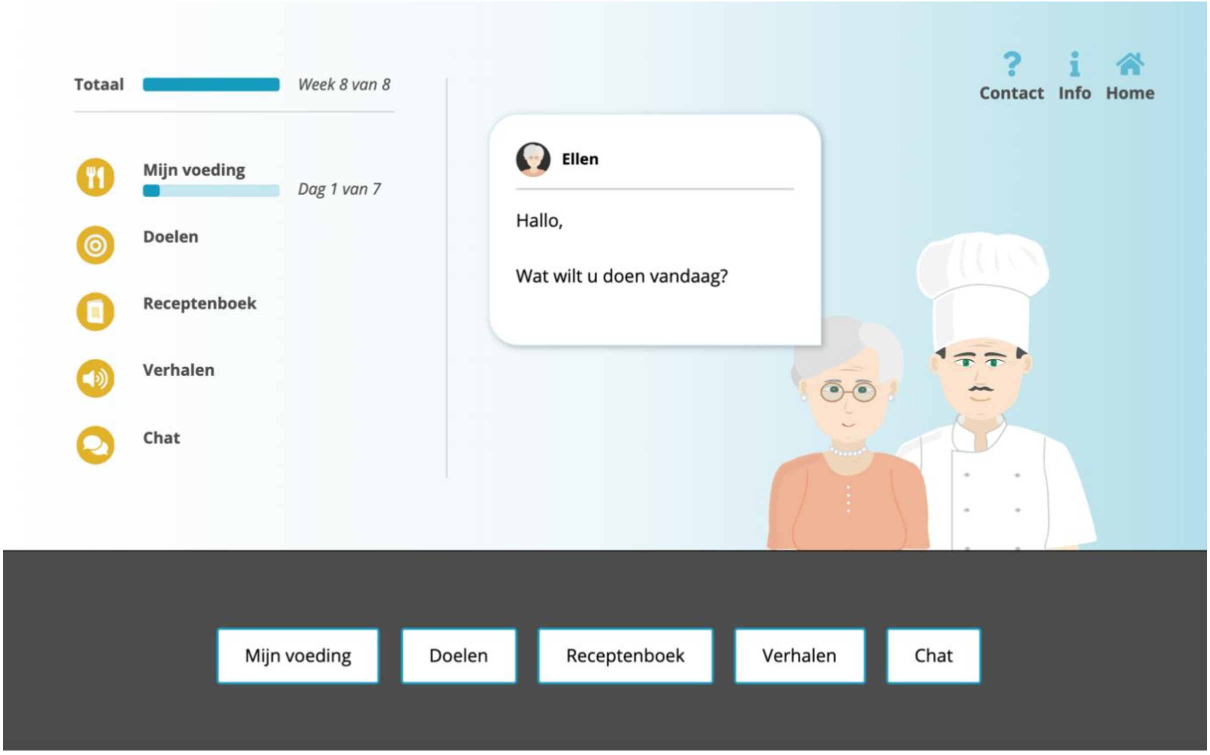

Inleiding

Geachte heer/mevrouw,

Wij vragen u om mee te doen aan een medisch-wetenschappelijk onderzoek.
Meedoen is vrijwillig. Om mee te doen is wel uw toestemming nodig.

Voordat u beslist of u wilt meedoen aan dit onderzoek, krijgt u uitleg over wat het onderzoek inhoudt. Lees deze informatie rustig door en vraag de onderzoeker uitleg als u vragen heeft. U kunt ook de onafhankelijk deskundige, die aan het eind van deze brief genoemd wordt, om aanvullende informatie vragen. U kunt er ook over praten met uw partner, vrienden of familie.
Algemene informatie over meedoen aan zo'n onderzoek vindt u op de website van de Rijksoverheid: www.rijksoverheid.nl/mensenonderzoek.

1. Algemene informatie

Dit onderzoek wordt gedaan door Wageningen University & Research, in samenwerking met het Nationaal Ouderenfonds, WAAG en Roessingh Research and Development. Voor dit onderzoek zijn we op zoek naar 60 proefpersonen.

De medisch-ethische toetsingscommissie METC-WU heeft dit onderzoek goedgekeurd. Algemene informatie over de toetsing van onderzoek vindt u op de website van de Rijksoverheid: www.rijksoverheid.nl/mensenonderzoek.

2. Doel van het onderzoek

In dit onderzoek gaat u de PACO-applicatie (app) gebruiken. Deze app is bedoeld om u te motiveren op het gebied van voedingsgedrag (zoals bewust eten, genieten van het eten, en voldoende groente eten) en sociale activiteiten (wat betrekking heeft op gevoelens van eenzaamheid en het aantal sociale contacten). Het doel van dit onderzoek is om te achterhalen hoe de app wordt gebruikt, wat de gebruikservaringen zijn en of de app gezond gedrag en sociale activiteiten kan stimuleren. De resultaten van dit onderzoek gaan we publiceren in wetenschappelijk tijdschriften en tijdens bijeenkomsten.

3. Achtergrond van het onderzoek

Uit wetenschappelijke literatuur is gebleken dat er nog te weinig bekend is over het gebruik en de toegevoegde waarde van virtuele coaches (u kunt deze beschouwen als digitale gesprekspartners). Het is belangrijk om hier inzicht in te verkrijgen. Zo kunnen we deze kennis verspreiden en toepassen in bestaande of nieuwe elektronische toepassingen.

4. Wat meedoen inhoudt

Als u meedoet, duurt dat totaal 8 tot 12 weken voor u. Als u meedoet, vragen we u om de app te gebruiken. Tijdens het gebruik van de PACO-app doorloopt u een 8-weeks programma, samen met twee virtuele coaches. Op de eerste pagina heeft u de virtuele coaches kunnen zien. U kunt met de coaches in dialoog over uw voedingsgedrag en sociale activiteiten. U bepaalt zelf hoe vaak u het

dialoog aan gaat met de coaches en op welk moment. Juist de keuzes die u daarin maakt zijn voor de onderzoekers interessant. De eerste week wordt u gevraagd om bij te houden wat u heeft gegeten. Tijdens de andere 7 weken is het programma flexibel. Er is een onderdeel waar u doelen kunt kiezen en zetten, op het gebied van gezondheid en sociale activiteiten. Ook hier bepaalt u zelf weer aan welke doelen u werkt, er wordt u dus niks opgelegd. Ook is er een handige zoekfunctie voor het zoeken naar een recept, er zijn verhalen over sociale activiteiten van andere ouderen, en er is een chat waar u met de andere deelnemers in contact kunt komen.

De helft van de deelnemers wordt willekeurig ingedeeld in groep 1, de andere helft van de deelnemers wordt willekeurig ingedeeld in groep 2. Indien u wordt ingedeeld in groep 1, ontvangt u direct de PACO-app. Indien u wordt ingedeeld in groep 2, vult u een extra vragenlijst in en ontvangt u na 4 weken de PACO-app. Het invullen van deze vragenlijst duurt 10 tot 20 minuten. Afhankelijk hiervan duurt uw deelname dus 8 of 12 weken.

Naast het gebruik van de app bestaat de studie uit de volgende onderdelen:
- Het thuis online invullen van drie of vier vragenlijsten: U ontvang een mail met daarin een link naar de vragenlijst. Het invullen duurt tussen de 10 en 30 minuten per vragenlijst. De vragen gaan over uw ervaring met betrekking tot de PACO-app, en uw gezondheid.
- Optioneel is een afsluitend interview van 30 minuten, waarin wij graag dieper zouden ingaan op uw ervaring. Dit interview kan telefonisch of bij u thuis, afhankelijk van uw voorkeur. Het interview zal door middel van audioapparatuur worden opgenomen.

Wanneer u besluit om deel te nemen en het toestemmingsformulier is ontvangen door de onderzoekers, gaat de studie van start. U ontvangt binnen enkele werkdagen per mail een bevestiging van uw deelname, en informatie over de groep waarin u bent ingedeeld. Ook geven wij u de nodige informatie over het gebruik van de applicatie.

U kunt altijd contact met ons opnemen, via de app, telefonisch of per mail. Indien u langer dan een week de app niet gebruikt, zullen wij contact met u opnemen om te vragen of er problemen zijn en of wij u ergens mee kunnen helpen.

5. Wat wordt er van u verwacht

Om het onderzoek goed te laten verlopen, is het belangrijk dat u zich aan de volgende afspraken houdt.

De afspraken zijn dat u:

- gebruik maakt van de PACO-app; en
- de vragenlijsten invult.

Het is belangrijk dat u contact opneemt met de onderzoeker:

- als u niet meer wilt meedoen aan het onderzoek.
- als uw contactgegevens wijzigen.

6. Mogelijke voor- en nadelen

Het is belangrijk dat u de mogelijke voor- en nadelen goed afweegt voordat u besluit mee te doen. Het gebruik van de PACO-applicatie kan inzage geven in uw gezondheidsgedrag, maar zeker is dat niet. Uw deelname draagt wel bij aan meer kennis over het gebruik van applicaties. Er zijn, voor zover bekend, geen nadelen van meedoen aan het onderzoek.

Deelname aan het onderzoek betekent wel:

- dat het een tijdsinvestering van u vraagt;
- dat u afspraken heeft waaraan u zich moet houden.

Al deze zaken zijn hiervoor onder punt 4, 5 en 6 beschreven.

7. Als u niet wilt meedoen of wilt stoppen met het onderzoek

U beslist zelf of u meedoet aan het onderzoek. Deelname is vrijwillig.

Als u wel meedoet, kunt u zich altijd bedenken en toch stoppen, ook tijdens het onderzoek. De gegevens die tot dat moment zijn verzameld, worden gebruikt voor het onderzoek. Indien u uw gegevens wilt laten verwijderen, is dit ook mogelijk. Dit kunt u aangeven bij de onderzoeker.

8. Einde van het onderzoek

Uw deelname aan het onderzoek stopt als

- alle bijeenkomsten zoals beschreven onder punt 4 voorbij zijn
- u zelf kiest om te stoppen
- de onderzoeker het beter voor u vindt om te stoppen
- Wageningen University, de overheid of de beoordelende medisch-ethische toetsingscommissie, besluit om het onderzoek te stoppen.

Het hele onderzoek is afgelopen als alle deelnemers klaar zijn.

Na het verwerken van alle gegevens informeert de onderzoeker u over de belangrijkste uitkomsten van het onderzoek. Dit gebeurt ongeveer een half jaar na uw deelname.

9. Gebruik en bewaren van uw gegevens

Voor dit onderzoek worden uw persoonsgegevens gebruikt en bewaard. Het gaat om gegevens zoals uw naam, adres, leeftijd en antwoorden op de vragenlijsten. Al uw gegevens worden verzameld en bewaard door Wageningen University & Research. Gegevens over hoe u de applicatie gebruikt worden automatisch verzameld door Roessingh Research and Development, maar ook deze gegevens worden na afloop van de studie enkel bewaard door Wageningen University & Research.

Vertrouwelijkheid van uw gegevens

Om uw privacy te beschermen krijgen uw gegevens een code. Uw naam en andere gegevens die u direct kunnen identificeren worden daarbij weggelaten. Alleen met de sleutel van de code zijn

gegevens tot u te herleiden. De sleutel van de code blijft veilig opgeborgen bij Wageningen University & Research. In rapporten en publicaties over het onderzoek zijn de gegevens niet tot u te herleiden.

Toegang tot uw gegevens voor controle

Sommige personen kunnen op de onderzoekslocatie toegang krijgen tot al uw gegevens. Ook tot de gegevens zonder code. Dit is nodig om te kunnen controleren of het onderzoek goed en betrouwbaar is uitgevoerd. Personen die ter controle inzage krijgen in uw gegevens zijn: drie onderzoekers van Wageningen University (Lean Kramer, Emely de Vet en Bob Mulder), de commissie die de veiligheid van het onderzoek in de gaten houdt, een monitor die voor de onderzoeker werkt, nationale en internationale toezichthoudende autoriteiten, waaronder de Inspectie Gezondheidszorg en Jeugd. Zij houden uw gegevens geheim. Wij vragen u voor deze inzage toestemming te geven.

Bewaartermijn gegevens

Uw gegevens moeten 10 jaar worden bewaard op de onderzoekslocatie.

Bewaren en gebruik van gegevens

Uw gegevens kunnen na afloop van dit onderzoek ook nog van belang zijn voor ander wetenschappelijk onderzoek op het gebied van gezondheidsapplicaties. Daarvoor zullen uw gegevens 10 jaar worden bewaard. U kunt op het toestemmingsformulier aangeven of u hier wel of niet mee instemt. Indien u hier niet mee instemt, kunt u gewoon deelnemen aan het huidige onderzoek.

Intrekken toestemming

U kunt uw toestemming voor gebruik van uw persoonsgegevens altijd weer intrekken. Dit geldt voor dit onderzoek en ook voor het bewaren en het gebruik voor het toekomstige onderzoek. De onderzoeksgegevens die zijn verzameld tot het moment dat u uw toestemming intrekt worden nog wel gebruikt in het onderzoek.

Meer informatie over uw rechten bij verwerking van gegevens

Voor algemene informatie over uw rechten bij verwerking van uw persoonsgegevens kunt u de website van de Autoriteit Persoonsgegevens raadplegen.

Bij vragen over uw rechten kunt u contact opnemen met de verantwoordelijke voor de verwerking van uw persoonsgegevens. Voor dit onderzoek is dat: Wageningen University.
Zie bijlage A voor contactgegevens.

Bij vragen of klachten over de verwerking van uw persoonsgegevens raden we u aan eerst contact op te nemen met de onderzoekslocatie. U kunt ook contact opnemen met de Functionaris voor de Gegevensbescherming van de instelling [zie bijlage A] of de Autoriteit Persoonsgegevens.

Registratie van het onderzoek

Informatie over dit onderzoek is ook opgenomen in een overzicht van medisch-wetenschappelijke onderzoeken namelijk https://www.toetsingonline.nl/to/ccmo_search.nsf/Searchform?OpenForm.

Daarin zijn geen gegevens opgenomen die naar u herleidbaar zijn. Na het onderzoek kan de website een samenvatting van de resultaten van dit onderzoek tonen. U vindt dit onderzoek onder ‘PACO’.

10. Verzekering voor proefpersonen

Voor iedereen die meedoet aan dit onderzoek is een verzekering afgesloten. De verzekering dekt schade door het onderzoek. Niet alle schade is gedekt. In **bijlage B** vindt u meer informatie over de verzekering en de uitzonderingen. Daar staat ook aan wie u schade kunt melden.

11. Geen vergoeding voor meedoen

Gebruik van de PACO applicatie kost u niets. U wordt niet betaald voor het meedoen aan dit onderzoek.

12. Heeft u vragen?

Bij vragen kunt u contact opnemen met Lean Kramer. Voor onafhankelijk advies over meedoen aan dit onderzoek kunt u terecht bij de onafhankelijke deskundige Monique Simons. Zij weet veel over het onderzoek, maar heeft niets te maken met dit onderzoek.
Indien u klachten heeft over het onderzoek, kunt u dit bespreken met de onderzoeker. Wilt u dit liever niet, dan kunt u zich wenden tot de onafhankelijk expert of klachtenfunctionaris. Alle gegevens vindt u in **bijlage A**: Contactgegevens.

13. Ondertekening toestemmingsformulier

Wanneer u voldoende bedenktijd heeft gehad, wordt u gevraagd te beslissen over deelname aan dit onderzoek. Indien u toestemming geeft, zullen wij u vragen deze op de bijbehorende toestemmingsverklaring schriftelijk te bevestigen. Door uw schriftelijke toestemming geeft u aan dat u de informatie heeft begrepen en instemt met deelname aan het onderzoek.
Zowel uzelf als de onderzoeker ontvangen een getekende versie van deze toestemmingsverklaring.

Dank voor uw aandacht.

Toestemmingsformulier

- Ik heb de informatiebrief gelezen. Ook kon ik vragen stellen. Mijn vragen zijn voldoende beantwoord. Ik had genoeg tijd om te beslissen of ik meedoe.

- Ik weet dat meedoen vrijwillig is. Ook weet ik dat ik op ieder moment kan beslissen om toch niet mee te doen of te stoppen met het onderzoek. Daarvoor hoef ik geen reden te geven.

- Ik geef toestemming voor het verzamelen en gebruiken van mijn gegevens voor de beantwoording van de onderzoeksvraag in dit onderzoek.

- Ik weet dat voor de controle van het onderzoek sommige mensen toegang tot al mijn gegevens kunnen krijgen. Die mensen staan vermeld in deze informatiebrief. Ik geef toestemming voor die inzage door deze personen.

  Ik geef □ **wel**

  □ **geen**

  toestemming om mijn persoonsgegevens langer te bewaren en te gebruiken voor toekomstig onderzoek op het gebied van gezondheidsapplicaties.

- Ik wil meedoen aan dit onderzoek.

Naam proefpersoon:

Handtekening: Datum: __ / __ / __

Ik verklaar dat ik deze proefpersoon voldoende heb geïnformeerd over het genoemde onderzoek. Als er tijdens het onderzoek informatie bekend wordt die de toestemming van de proefpersoon zou kunnen beïnvloeden, dan breng ik hem/haar daarvan tijdig op de hoogte.

Naam onderzoeker: Lean Kramer

Handtekening: Datum: __ / __ / __
