# Supplementary material for: Use and Effect of Web-Based Embodied Conversational Agents for Improving Eating Behavior and Decreasing Loneliness Among Community-Dwelling Older Adults: Protocol for a Randomized Controlled Trial
Source: JMIR Res Protoc. 2021 Jan 6;10(1):e22186. doi: 10.2196/22186 (PMC7817356; doi:10.2196/22186)
Supplement: Multimedia Appendix 2 [file resprot_v10i1e22186_app2.pdf]

Subsidieprogramma / Subsidy programme	:	**Create Health**
Dossiernummer / Dossier number	:	**40-44300-98-110**
Aanvrager / applicant	:	**Prof. dr. E. de Vet PhD**
Projecttitel / Project title	:	**How to design Persuasive E-health Agents for Coaching Older adults towards dietary behavior change (PACO)?**
Beoordelingscode / Assessment code	:	**B.2017.00F90**

1. Introduction

Proposals for the Create Health programme should be judged according to the criteria as described in the assessment form. It is essential that you take all these criteria into consideration. Always provide stronger and weaker points for each of these criteria (a SWOT analysis). ***Please check the Create Health call text on page 5 and 6 for a description of the criteria.*** *An in depth explanation accompanying your critiques/positive feedback, thus enticing a written debate with the applicants is essential. Even if you are very positive a critical evaluation remains necessary in order for the committee to judge the value of the research proposal. Superficial evaluations do not provoke applicants to defend or elaborate on their ideas and plans (through rebuttal) and are of little use to the committee.*

Your assessment will be treated confidentially. Please, do not refer to your identity. The assessment will (anonymously) be made available to the applicant, who is invited to give a written rebuttal which will be sent to the programme committee.

2. Quality

Legenda: S (Sufficient), I (Insufficient)

2.1 Innovative and scientific quality and clarity

S	I
X	

Consider the following factors:
- Originality / innovativeness of the project;
- Clarity of the problem definition and the proposed research method;
- Scientific quality of the proposed study, method appropriate to the goal of the project.
- The proposal must involve experimental or theoretical activities ('fundamental research') performed primarily for the acquisition of new knowledge of fundamental aspects of the development or use of e-health under the three themes, without any direct commercial application or use in view;

Please justify your score by commenting on the strengths and weaknesses of the proposal on this criterium.
- *Strengths*
- *weaknesses*

The proposed project addresses a societal problem of overweight (and loneliness) in older adults. By developing a virtual agents to deliver the information regarding weight loss, the authors aim to better reach the lower educated who have more difficulty to process text-based health information.

There is a scientific need for more understanding on how to design persuasive virtual agents in healthcare settings.

The authors aim to include the end-user in the development of their virtual agent via a participatory design approach. This allows them to tailor the agent and its messages to best-practices throughout the project.

The project outcomes will inform a wider audience of researchers and developers in the field of virtual agents in healthcare settings.

The project is that research on long-term technology use in general is scarce, and thus even more so for virtual or embodied agents.

Even though to date the main approach in healthcare might still be text-based information, the current project proposal is absolutely not the first to introduce a persuasive virtual agent in eHealth. There have been many similar projects -even for weight loss specifically- as early as the 1960s. I highly recommend the authors to take the results of these earlier projects, both on virtual as well as embodied agents, into account. The authors

accurately state that these earlier projects only discuss their theoretical implications in very limited ways and so far have produced minor fundamental / theoretical progress.

The theoretical foundation of the proposal is not very convincing. The authors plan to rely on the ELM. Although incorporating such a theory allows for minor rationalization of health messages to specific target groups, this theory only includes an attitude change (which the authors acknowledge). Even more problematic is that the hard-to-reach lower educated target group (which the authors stress are more at risk for overweight) are more likely to take the peripheral route, which results in only minimal effects -if any- and definitely no long-term changes in attitudes. The ideas the authors point out for needed behavior change are presented as a list of (random?) keywords and lack a strong theoretical foundation and integration into the proposed project.

2.2 Added value, including generalisability and reproducibility

S	I
X	

Added value, including generalisability and reproducibility Consider the following factors:

- Clear description of all existing innovations and studies in the field in question and what the proposed project will add to this;
- Reproducibility, applicability and generalisability of the project;
- Addresses knowledge transfer/consolidation of the expected results on a proper timescale;
- Involvement of envisaged users at an early stage to ensure optimum accessibility.

Please justify your score by commenting on the strengths and weaknesses of the proposal on this criterium.

- *Strengths*
- *weaknesses*

The authors describe several of their own earlier related projects and clearly describe how to current project will incorporate these previous findings and further addresses open questions.

The authors aim for a wider outreach for their results that stretch beyond the scientific community. For example, they aim to reach developers and practitioners by transforming their scientific output into a manual of design guidelines. Additionally, I am very pleased to read that authors are planning to share their data set(s) at the end of the project.

The bottom-up approach via participatory design will enable to authors to adjust the development of their virtual agent to the demands and wishes of the end-users. This will facilitate the establishment of a persuasive health virtual agent that is both acceptable and successful.

Throughout the whole application, I was not fully convinced whether the persuasive model developed in WP2 will be grounded in a theoretical basis. In addition, the outcome variable of the project -which is labeled as acceptance, an extremely broad term with infinite independent factors- remains extremely vague and needs further quantification as well as qualification.

2.3 Suitability and quality of action plan

S	I
X	

Consider the following factors:

- Realistic time schedule;
- Coherence between the work packages;
- Feasible milestones and deliverables;
- Effective approach to research.

Please justify your score by commenting on the strengths and weaknesses of the proposal on this criterium.

- *Strengths*
- *weaknesses*

The work plan is written in detail and follows a logical approach to obtaining the project goals. The first step (WP1) is extensive with multiple iterations to develop a list of relevant factors via focus groups with end-users. As a second step (WP2), the authors proposal to run several experiments with proto-types to test the effectiveness of these factors which will produce input to develop the virtual agent. Based on this input, the authors propose to update the content and interaction strategies of their existing virtual agent (WP3), which will be tested with end-users over a longer period (WP4) with both quantitative and qualitative data as well as objective log data. The final step described the authors plans for knowledge dissemination which aims to reach beyond the scientific community (WP5). The timeline for this project sounds feasible.

2.4 Partners/allocation of work

S	I
X	

Partners/allocation of work Consider the following factors:

- Composition of the consortium. The project must be performed in a public-private partnership consisting of at least:
    1. research organisation active in healthcare/welfare,
    2. research organisation active in the creative industries,
    3. target group (organisations representing patients, clients or elderly people)
    4. undertaking.
- Co-ordination of synergetic activities between partners;
- Excellence and expertise of the consortium partners in relation to the project;
- Added value of the consortium partners to the quality of the project;
- The target group must be involved at all stages of the project as an equal partner in the consortium. Projects should address issues that arise in daily practice. The project group should therefore demonstrably involve people with individual and professional experience of the theme in question, in both the preparation and the implementation of the project. All proposals must describe how experience experts can best participate and what form participation by end users should take.

Please justify your score by commenting on the strengths and weaknesses of the proposal on this criterium.

- *Strengths*
- *weaknesses*

The consortium exists of a strong interdisciplinary group of both researchers and practitioners with expertise in the relevant related areas included in the proposal.

3. Overall quality assessment

Legenda: E (Excellent), VG (Verry Good), G (Good), S (Satisfactory), U (Unsatisfactory)

3.1 Overall quality assessment

E	VG	G	S	U
		X		

Please justify your final assessment by summarising or briefly commenting on the strengths and weaknesses of the proposal.

Overall, the authors propose an interesting and contemporary project that addresses a societal problem. However, especially because they stress that their main aim is to perform fundamental research that adds a theoretical understanding to the field of virtual agents, I would have expected a more profound theoretical framework to ground their research project.

4. Budget

Legenda: TH (Too high), R (realistic), TL (too low)

4.1 Budget

TH	R	TL
	X	

Please explain your score:

X

Subsidieprogramma / Subsidy programme	:	**Create Health**
Dossiernummer / Dossier number	:	**40-44300-98-110**
Aanvrager / applicant	:	**Prof. dr. E. de Vet PhD**
Projecttitel / Project title	:	**How to design Persuasive E-health Agents for Coaching Older adults towards dietary behavior change (PACO)?**
Beoordelingscode / Assessment code	:	**B.2017.00F91**

1. Introduction

Proposals for the Create Health programme should be judged according to the criteria as described in the assessment form. It is essential that you take all these criteria into consideration. Always provide stronger and weaker points for each of these criteria (a SWOT analysis). ***Please check the Create Health call text on page 5 and 6 for a description of the criteria.*** *An in depth explanation accompanying your critiques/positive feedback, thus enticing a written debate with the applicants is essential. Even if you are very positive a critical evaluation remains necessary in order for the committee to judge the value of the research proposal. Superficial evaluations do not provoke applicants to defend or elaborate on their ideas and plans (through rebuttal) and are of little use to the committee.*

Your assessment will be treated confidentially. Please, do not refer to your identity. The assessment will (anonymously) be made available to the applicant, who is invited to give a written rebuttal which will be sent to the programme committee.

2. Quality

Legenda: S (Sufficient), I (Insufficient)

2.1 Innovative and scientific quality and clarity

S	I
X	

Consider the following factors:

- Originality / innovativeness of the project;
- Clarity of the problem definition and the proposed research method;
- Scientific quality of the proposed study, method appropriate to the goal of the project.
- The proposal must involve experimental or theoretical activities ('fundamental research') performed primarily for the acquisition of new knowledge of fundamental aspects of the development or use of e-health under the three themes, without any direct commercial application or use in view;

Please justify your score by commenting on the strengths and weaknesses of the proposal on this criterium.

- *Strengths*
- *weaknesses*

Innovative in both the virtual agent and targeting the older adult.
Is basically a feasibility study with no controls.

2.2 Added value, including generalisability and reproducibility

S	I
X	

Added value, including generalisability and reproducibility Consider the following factors:

- Clear description of all existing innovations and studies in the field in question and what the proposed project will add to this;
- Reproducibility, applicability and generalisability of the project;
- Addresses knowledge transfer/consolidation of the expected results on a proper timescale;
- Involvement of envisaged users at an early stage to ensure optimum accessibility.

Please justify your score by commenting on the strengths and weaknesses of the proposal on this criterium.

- *Strengths*
- *weaknesses*

Clear description of what has been developed and what still needs to be developed as well as the investigators role in formative work.
Timeframe appears appropriate if I am reading this clearly, a little over 2 years.

2.3 Suitability and quality of action plan

S	I
X	

Consider the following factors:

- Realistic time schedule;
- Coherence between the work packages;
- Feasible milestones and deliverables;
- Effective approach to research.

Please justify your score by commenting on the strengths and weaknesses of the proposal on this criterium.

- *Strengths*
- *weaknesses*

yes, realistic, appropriate milestones provided.

2.4 Partners/allocation of work

S	I
X	

Partners/allocation of work Consider the following factors:

- Composition of the consortium. The project must be performed in a public-private partnership consisting of at least:
    1. research organisation active in healthcare/welfare,
    2. research organisation active in the creative industries,
    3. target group (organisations representing patients, clients or elderly people)
    4. undertaking.
- Co-ordination of synergetic activities between partners;
- Excellence and expertise of the consortium partners in relation to the project;
- Added value of the consortium partners to the quality of the project;
- The target group must be involved at all stages of the project as an equal partner in the consortium. Projects should address issues that arise in daily practice. The project group should therefore demonstrably involve people with individual and professional experience of the theme in question, in both the preparation and the implementation of the project. All proposals must describe how experience experts can best participate and what form participation by end users should take.

Please justify your score by commenting on the strengths and weaknesses of the proposal on this criterium.

- *Strengths*
- *weaknesses*

Yes, experienced team. They have had a related grant before and work together productively.

3. Overall quality assessment

Legenda: E (Excellent), VG (Verry Good), G (Good), S (Satisfactory), U (Unsatisfactory)

3.1 Overall quality assessment

E	VG	G	S	U
X				

Please justify your final assessment by summarising or briefly commenting on the strengths and weaknesses of the proposal.

This is a very clear and realistic proposal. The team is experienced and the milestones seem entirely feasible.

4. Budget

Legenda: TH (Too high), R (realistic), TL (too low)

4.1 Budget

TH	R	TL
	X	

Please explain your score:

I don't have a good background on these costs but they do not seem unrealistic.

Subsidieprogramma / Subsidy programme	:	**Create Health**
Dossiernummer / Dossier number	:	**40-44300-98-110**
Aanvrager / applicant	:	**Prof. dr. E. de Vet PhD**
Projecttitel / Project title	:	**How to design Persuasive E-health Agents for Coaching Older adults towards dietary behavior change (PACO)?**
Beoordelingscode / Assessment code	:	**B.2017.00F92**

1. Introduction

Proposals for the Create Health programme should be judged according to the criteria as described in the assessment form. It is essential that you take all these criteria into consideration. Always provide stronger and weaker points for each of these criteria (a SWOT analysis). ***Please check the Create Health call text on page 5 and 6 for a description of the criteria.*** *An in depth explanation accompanying your critiques/positive feedback, thus enticing a written debate with the applicants is essential. Even if you are very positive a critical evaluation remains necessary in order for the committee to judge the value of the research proposal. Superficial evaluations do not provoke applicants to defend or elaborate on their ideas and plans (through rebuttal) and are of little use to the committee.*

Your assessment will be treated confidentially. Please, do not refer to your identity. The assessment will (anonymously) be made available to the applicant, who is invited to give a written rebuttal which will be sent to the programme committee.

2. Quality

Legenda: S (Sufficient), I (Insufficient)

2.1 Innovative and scientific quality and clarity

S	I
X	

Consider the following factors:

- Originality / innovativeness of the project;
- Clarity of the problem definition and the proposed research method;
- Scientific quality of the proposed study, method appropriate to the goal of the project.
- The proposal must involve experimental or theoretical activities ('fundamental research') performed primarily for the acquisition of new knowledge of fundamental aspects of the development or use of e-health under the three themes, without any direct commercial application or use in view;

Please justify your score by commenting on the strengths and weaknesses of the proposal on this criterium.

- *Strengths*
- *weaknesses*

The project presents an approach to promoting a healthy diet among elderly that is highly innovative. Having a virtual coach has been shown in other health fields to result in similar adherence levels as face-to-face solutions, thus providing a cost-effective, optimized approach for eHealth interventions.

The project includes fundamental research on the peripheral route (studying needs regarding messages cues such as appearance, rather than the content of the messages). It includes qualitative and survey research, fitting with the tight time schedule of the project. The project furthermore includes a real-life evaluation on acceptance of the virtual coach.

The focus of the project is not to study the effectiveness of a virtual coach in promoting a healthy diet, but the feasibility and acceptability among elderly to use such a tool.

A weakness may lie in some methodological vagueness. For example, WP2 includes experiments, in some places referred to as qualitative experiments and some as quantitative. It is unclear if these are considered as true experiments with a control condition, and what this condition would consist of. Sample size is described as 'large' but it would be good to have more information on sampling strategy, inclusion criteria (65y and above?) and exact size to get a grasp on representativeness and generalizability of findings. Another weakness may be the uncertainty of how the disease area 'prevention of loneliness in frail elderly' will be addressed by the project, that is heavily focused on prevention of overweight. It is unclear if the presence of a virtual coach itself is expected to

reduce feelings of loneliness, or if the virtual coach will also include behavior change strategies to prevent loneliness in the message content.

Strengths are clearly that the project employs a variety of research methods that are feasible within the project time and will lead to novel insights on this topic, and can be a first and necessary stepping stone to further research investigating effectiveness of such a tool. The research approach is in line with state-of-the-art methods (e.g. co-design, data logs) and can be expected to be highly innovative both in the fundamental scientific and applied field.

2.2 Added value, including generalisability and reproducibility

S	I
X	

Added value, including generalisability and reproducibility Consider the following factors:
- Clear description of all existing innovations and studies in the field in question and what the proposed project will add to this;
- Reproducibility, applicability and generalisability of the project;
- Addresses knowledge transfer/consolidation of the expected results on a proper timescale;
- Involvement of envisaged users at an early stage to ensure optimum accessibility.

Please justify your score by commenting on the strengths and weaknesses of the proposal on this criterium.
- *Strengths*
- *weaknesses*

The project has given a good overview of existing innovations in this field, it builds on an existing tool and European project expertise. The project findings can be expected to be generalisable to other fields, given its focus on the peripheral route, feasibility and acceptability of such a tool, rather than the specific message content pertaining to one health domain. Knowledge transfer is envisaged on a sufficiently wide scale. Users are involved from the start via the co-design group and throughout the project.

A slight concern here may be the size of the co-design panel (2-4 members), which may be small to represent views of a varied target group (active seniors, disabled, single, etc, and their family and carers) and the project partners may consider to increase the size or use several co-design panel groups.

The project will use a tablet pc for testing, but it would be good to know if the solution can also run on other devices (e.g. website, computer, Phone). If it appears during project execution that few elderly use or possess tablets, this would be a barrier in further generalizability of this tool.

2.3 Suitability and quality of action plan

S	I
X	

Consider the following factors:
- Realistic time schedule;
- Coherence between the work packages;
- Feasible milestones and deliverables;
- Effective approach to research.

Please justify your score by commenting on the strengths and weaknesses of the proposal on this criterium.
- *Strengths*
- *weaknesses*

The project appears very strong in presenting a realistic, coherent, feasible and effective approach.
A quick integration of the ideas from WP1 in the different versions that will be tested in WP2, may be challenging but can be considered feasible given the expertise of the consortium partners in such development.

2.4 Partners/allocation of work

S	I
X	

Partners/allocation of work Consider the following factors:
- Composition of the consortium. The project must be performed in a public-private partnership consisting of at least:
    1. research organisation active in healthcare/welfare,
    2. research organisation active in the creative industries,
    3. target group (organisations representing patients, clients or elderly people)
    4. undertaking.

- Co-ordination of synergetic activities between partners;
- Excellence and expertise of the consortium partners in relation to the project;
- Added value of the consortium partners to the quality of the project;
- The target group must be involved at all stages of the project as an equal partner in the consortium. Projects should address issues that arise in daily practice. The project group should therefore demonstrably involve people with individual and professional experience of the theme in question, in both the preparation and the implementation of the project. All proposals must describe how experience experts can best participate and what form participation by end users should take.

Please justify your score by commenting on the strengths and weaknesses of the proposal on this criterium.
- *Strengths*
- *weaknesses*

Each partner brings complementary and necessary expertise. The activities appear well co-ordinated between partners. Their expertise with co-design and participation of the target group is evident from the project proposal and their experience.

3. Overall quality assessment

Legenda: E (Excellent), VG (Verry Good), G (Good), S (Satisfactory), U (Unsatisfactory)

3.1 Overall quality assessment

E	VG	G	S	U
X				

Please justify your final assessment by summarising or briefly commenting on the strengths and weaknesses of the proposal.

The project proposes an innovative, feasible, generalizable approach that will optimize current eHealth solutions and can address the issues of healthy ageing.
The comments raised during this project review arise from a need for further specification and detail, and can be expected to be addressed during a rebuttal. None are major or detrimental to the project's success.

4. Budget

Legenda: TH (Too high), R (realistic), TL (too low)

4.1 Budget

TH	R	TL
	X	

Please explain your score:

Given the use of an existing tool, the focus on feasibility and acceptability rather than effectiveness of behavior change, and the available expertise of the partners, the project budget can be considered realistic.

Projectnummer: 40-44300-98-110

Naam projectleider: Prof. dr. E. de Vet

Projecttitel: How to design Persuasive E-health Agents for Coaching Older adults towards dietary behavior change (PACO)?

Samenvattende reactie

We thank all three reviewers for reading our proposal and providing valuable feedback. We were very pleased to read that all three reviewers were positive about the innovative character, the quality and merit of our project proposal, as well as the feasibility (2 reviewers scored the proposal as excellent, one as good). Some requests for clarification were made about the theoretical foundation for our work, and the methodological setup of WP 2. We are pleased to have the opportunity to clarify some of these issues, which we could not fully elaborate on in the proposal due to word limits. We provide these clarifications in our responses to the individual reviewers.

Reactie op referent met Beoordelingscode: B.2017.00F90

The reviewer found the proposal interesting and contemporary, with a sound and feasible workplan conducted by a strong interdisciplinary group. The reviewer asked questions about the theoretical framework that underlies the work in WP1 and WP2. As the reviewer also seems to acknowledge, and which provided the rationale for initiating this proposal, there is currently a lack of fundamental understanding of the effective elements of eHealth communication through virtual agents and its working mechanisms. Hence, inspired by existing theories, we seek to contribute to the state of the art research by *building* this fundamental theoretical insights rather than merely *applying* existing theories.

It is true that we see the Elaboration Likelihood Model as a general model that influences the direction of the project proposal (i.e., focusing on the 'soft' factors that make up a virtual agent's persuasiveness, instead of focusing on the strength and logic of its arguments) and that we did not provide a very detailed overview of the different theoretical models that are available in the literature, that describe how virtual agents can persuade people to commit to a healthier lifestyle, and how they shape the explorative phase in WP1. Rather, we named the most prominent and influential model of today only (Fogg's persuasive model), as we see this as the most important source of inspiration for the activities in WP1. In the execution of WP1 well-established theories such as Social Cognitive Theory (Bandura, 1991), the Transtheoretical Model (Prochaska & DiClemente, 1983), Theory of Planned Behavior (Ajzen, 1991), more state of the art models of behavior change in eHealth such as the Persuasive Systems Design model (Oinas-Kukkonen, 2009), as well as specific theories of Goal-Setting (Locke & Latham, 2002), and applied theories of tailored communication (op den Akker et al., 2014) provide the point of departure.

During the co-design sessions we will explore the role of the factors that play an important role in these theories (such as self-efficacy and the social norm). We would like to stress, however, that we do not see it as the goal of WP1 to confirm the importance of factors that have already been listed in previous theories (especially as they are not developed for the context of virtual avatars), but that we value the possibility to design these sessions with an open mind, so that we can also identify novel and unexpected factors. So, in sum, we apply a deductive as well as an inductive approach in gathering a set of factors that make up a virtual eHealth agent's persuasiveness. This way, the co-design sessions and the generation of the persuasive model in WP2 (that heavily hinges on the output of WP1) are inspired on a strong theoretical background, and a set of factors that are new and unique for the design context.

Next, the reviewer requested more clarification on our understanding of the term 'acceptance', which is an important target variable in our longitudinal evaluation study. We interpret acceptance as in the Technology Acceptance Model (Davis, 1989) and the Unified Theory of Acceptance and Use of Technology (Venkatesh, Morris, Davis and Davis, 2003) which posit that it is the behavioral intention to use a specific technology for a specific goal (the intention to use the virtual agent to help to adopt a healthier nutritional lifestyle in this case). This intention, as such, will be influenced by a predetermined set of factors (e.g., ease of use, usefulness) and, as we anticipate, be influenced by a set of factors that that has not been identified before. In WP1 and WP2 we will elicit these factors. As these technology acceptance models have been used in the past for a variety of novel technologies (either health-related or communication-related), using these models enables comparison in more quantitative terms of the acceptance of virtual agents compared to other technologies.

Reactie op referent met Beoordelingscode: B.2017.00F91

The referent characterizes our project proposal as innovative, clearly written, realistic, and written by an experienced and productive team. We appreciate the evaluation of the reviewer and based on the positive and constructive feedback, we have no further reply to provide.

Reactie op referent met Beoordelingscode: B.2017.00F92

We were pleased to read that according to the reviewer the proposal could contribute to innovative, feasible, generalizable insights to optimize eHealth solutions. The reviewer invited us to elaborate on the methodological setup of the experiments that will be conducted in WP2, as the distinction between the two different data collection approaches was insufficiently described. In WP2, we will conduct experiments in which different versions of a virtual agent are tested for acceptance. In this setup, we will not have an inactive control condition. Instead, we will implement different factors of the virtual agent in different versions (e.g., communication styles, content, visual features), to ascertain the importance of the design of these factors, and subsequently, measure the same variables through a questionnaire. As such the virtual agent will be adapted throughout the trial and evaluated in subsequent steps. This novel evaluation approach labelled as "sequential multiple assignment randomized trials", has been advocated especially for interventions and treatments that can be adapted and personalized rather rapidly (e.g. Kidwell & Hyde, 2016: Sherwood et al., 2016). The quantitative data gathered by the questionnaires will be triangulated by qualitative data, obtained from group interviews. This way, we can utilize the strength of both data collection methods. The large sample of older adults that will participate in the WP, consists of people of 65 years of age or older. We aspire to include at least 100 older adults for the quantitative data collection and 15 older adults in the group interviews. For both groups we will gather a sample that is representative of the Dutch population of older adults, aged 65 years and older.

Next, the reviewer asked us to explain how prevention of loneliness in frail elderly will be addressed during the project. We will focus the project predominantly on the prevention of overweight and adopting a healthy nutritional lifestyle (as introducing a dual focus whereby both foci are equally important, would result in the need to customize the virtual agent technology for both goals, which is not a feasible option given the project restraints in terms of time and money). The results of the project, nonetheless, will inform us how to design virtual agents that can successfully persuade an older adult to lead a healthier life, including persuading an older adult to make use of services that support the participation in social activities (as one aspect in the prevention of loneliness). This may include strategies that lead to healthier eating and reducing loneliness at the same time, such as persuading older adults to eat together.

The reviewer was slightly concerned about the size of the co-design panel, which we initially anticipated to be a group of 2 to 4 prospective end-users. The reviewer rightfully pointed out, however, that it might be wiser to increase the size of this group, so that every sub-group of this population can be included. We agree with him/her on this point and will include 10 persons who will be selected on their ability to represent different sub-groups within the total population of older adults.

A final question from this reviewer was how easily the virtual agent can run on other modalities than a Tablet PC (e.g., a smartphone, desktop PC or smart TV). The technological background framework provided by RRD is a Java-based client-server solution in which the client applications are written for Android. This means that the virtual agent works, without any alterations, on both tablets and mobile phones. Next, the underlying technology uses HTML/JavaScript for presenting its graphical user interfaces. This allows us to run the virtual agent – after some small modifications – to run in any web browser on a desktop PC or smart TV. This greatly enhances the chances of success of implementation in a real-life setting during the project and ensures that the project results (in terms of technology) can be easily exploited and scaled up.
